# Supplementary material for: Evaluation of the HEAL™ing Mental Health program: A prospective cohort study of short-term changes from a physical activity and lifestyle education program for people with mental health disorders living in rural Australia
Source: PLoS One. 2024 Mar 13;19(3):e0299859. doi: 10.1371/journal.pone.0299859 (PMC10936783; doi:10.1371/journal.pone.0299859)
Supplement: S1 Table — (DOCX) [file pone.0299859.s001.docx]

**S1 Table Stage of change and K-10 scores at baseline for included and excluded participants**

|  | **Included**  **N=99** | **Excluded**  **N=18** |
| --- | --- | --- |
| Stage of change | | |
| Pre-contemplation | 4 | 0 |
| Contemplation | 11 | 2 |
| Preparation | 28 | 5 |
| Action | 24 | 4 |
| Maintenance | 15 | 3 |
| Missing | 17 | 4 |
| K10 | | |
| 10-19: likely to be well | 53 | 9 |
| 20-24: likely to have a mild mental disorder | 21 | 3 |
| 25-29: likely to have a moderate mental disorder | 8 | 2 |
| 30-50: likely to have a severe mental disorder | 15 | 4 |
| missing | 2 | 0 |
