# Supplementary material for: Evaluation of the HEAL™ing Mental Health program: A prospective cohort study of short-term changes from a physical activity and lifestyle education program for people with mental health disorders living in rural Australia
Source: PLoS One. 2024 Mar 13;19(3):e0299859. doi: 10.1371/journal.pone.0299859 (PMC10936783; doi:10.1371/journal.pone.0299859)
Supplement: Attachment — Submitted filename: Response to Reviewers.docx [file pone.0299859.s003.docx]

9^th^ November 2023

Dear Editor,

Thank you for the positive comments on our submission *“Evaluation of the HEALTMing Mental Health program: a prospective cohort study of short-term changes from a physical activity and lifestyle education program for people with mental health disorders living in rural Australia”*

Below we outline the changes made in response to the reviewer and editorial requirements provided by email on 24 October 2023.

With respect to the sharing of a de-identified data set, unfortunately we are unable to do this as our data is owned by a third-party organization and the data contains identifiable information on the delivery sites and the participants. If you need to discuss this matter with the third-party organisation, I suggest you contact Dr Channa Marsh, HEAL Project Officer, Exercise and Sports Science Australia (ESSA) on +618 3171 9687.

All authors have approved the manuscript and its changes. We look forward to hearing from you soon.

With thanks, on behalf of my co-authors,

Annette J. Raynor (PhD)

Associate Professor Exercise and Sport Science

of the variance components and model parameters,

| **Reviewer comment** | | **Author response** |
| --- | --- | --- |
| 1. The first paragraph of introduction section could have a | | Thank you, this is a good suggestion. We have added the following sentence to the first paragraph:  “Health promoting interventions that work around these disadvantages are important for equitable health opportunities.” |
| link sentence at the end regarding the mental health | |  |
| and physical activity/lifestyle factors to indicate that | |  |
| what is coming in the second paragraph. However, the | |  |
| rest part of the introduction is well written. | |  |
| 2. Most of the references at the end of the sentences are | | Our apologies for this oversight – the formatting of references has been corrected. |
| after the punctuation (e.g., line # 38,45,51, 56, 58, etc.), | |  |
| which should be before the punctuations. | |  |
| 3. In the method section, the first mention of ESSA (line | | Our apologies for this – the acronyms have been corrected and spelled out at first use. Lines 85 (ESSA), 91 (CALD), 100 (AEPs). |
| 89), CALD (line 94) and AEP (line 102) were acronyms, | |  |
| not abbreviated earlier in the paper. | |  |
| 4. Line 176 to 178: the last part of the sentence is not clear to me, can you please rewrite it? | | Sorry for the confusion with this sentence. We have amended it to now read:  “All but two of the excluded participants had dropped out before completing the post- testing.” |
| "This attendance cut-off strengthens the complete data (data present before and after the intervention) as only two participants did not subsequently drop out of program completely" | |  |
| 5. | The use of restricted maximum likelihood (REML) estimation in Liner Mixed Model (LMM) is often preferred because it provides more accurate estimates | Thank you for providing this example of better justification of our choice. We have further explained as follows: |
|  | especially when the fixed effects and random effects are correlated. REML estimation aims to maximize the likelihood of the random effects while marginalizing over the fixed effects, making it a suitable choice for  many practical applications. If this the case to preferring this model in analysing this study data, in the methodology part (from 180 line onward) this should be explained. | “We chose REML as the way participants differ from one another may be related to their starting values. The REML enables more accurate estimates of the variance and model parameters when such correlation between fixed effects and random effects may be present. As only two time points were present, only random intercepts for participants were included as to not risk over fitting by also including random slopes.“ |

| **Additional requirements** | **Response** |
| --- | --- |
| 1. Please ensure that your manuscript meets PLOS | We have reviewed the journal formatting and |
| ONE's style requirements, including those for file | changed the following: |
| naming. The PLOS ONE style templates can be found at |  |
| https://journals.plos.org/plosone/s/file?id=wjVg/PLOSO | • Level one headings formatted to sentence case, |
| ne_formatting_sample_main_body.pdf and | bold, 18. |
| https://journals.plos.org/plosone/s/file?id=ba62/PLOSO |  |
| ne_formatting_sample_title_authors_affiliations.pdf | • Abstract sub-headings removed |
|  | • Table and figure headings and legends amended |
|  | • Merged cells in tables have been amended |
|  | • Supplementary files (and in-text references to supplementary files) have been renamed. |
|  | The author affiliations have the location added and the corresponding author is included on cover page. |
| 2. We note that you have indicated that data from this | Our apologies. |
| study are available upon request. PLOS only allows data |  |
| to be available upon request if there are legal or ethical | We are unable to share data as it contains |
| restrictions on sharing data publicly. For more | identifiable information on the delivery sites and |
| information on unacceptable data access restrictions, | participants and is owned by a third-party |
| please see <http://journals.plos.org/plosone/s/data-> | organization. |
| availability#loc-unacceptable-data-access-restrictions. |  |
| In your revised cover letter, please address the |  |
| following prompts: |  |
| a) If there are ethical or legal restrictions on sharing a |  |
| de-identified data set, please explain them in detail |  |
| (e.g., data contain potentially sensitive information, |  |
| data are owned by a third-party organization, etc.) and |  |
| who has imposed them (e.g., an ethics committee). |  |
| Please also provide contact information for a data |  |
| access committee, ethics committee, or other |  |
| institutional body to which data requests may be sent. |  |
| b) If there are no restrictions, please upload the |  |
| minimal anonymized data set necessary to replicate |  |
| your study findings as either Supporting Information |  |
| files or to a stable, public repository and provide us with |  |
| the relevant URLs, DOIs, or accession numbers. For a list |  |
| of acceptable repositories, please |  |
| see <http://journals.plos.org/plosone/s/data-> |  |
| availability#loc-recommended-repositories. |  |
| We will update your Data Availability statement on your |  |
| behalf to reflect the information you provide. |  |

| 3. We note that you have stated that you will provide repository information for your data at acceptance. Should your manuscript be accepted for publication, we will hold it until you provide the relevant accession numbers or DOIs necessary to access your data. If you wish to make changes to your Data Availability statement, please describe these changes in your cover letter and we will update your Data Availability statement to reflect the information you provide. | This was selected in error. We are unable to share data as it contains identifiable information on the delivery sites and participants and is owned by a third-party organization. |
| --- | --- |
| 4. Please include captions for your Supporting Information files at the end of your manuscript, and update any in-text citations to match accordingly. Please see our Supporting Information guidelines for more  information: <http://journals.plos.org/plosone/s/support> ing-information. | These have been updated |
| 5. Please review your reference list to ensure that it is complete and correct. If you have cited papers that have been retracted, please include the rationale for doing so in the manuscript text, or remove these references and replace them with relevant current references. Any changes to the reference list should be mentioned in the rebuttal letter that accompanies your revised manuscript. If you need to cite a retracted article, indicate the article’s retracted status in the References list and also include a citation and full reference for the retraction notice. | Thank you. The references have been reviewed. |
